# On macromolecular refinement at subatomic resolution with interatomic scatterers

**DOI:** 10.1107/S0907444907046148

**Published:** 2007-10-17

**Authors:** Pavel V. Afonine, Ralf W. Grosse-Kunstleve, Paul D. Adams, Vladimir Y. Lunin, Alexandre Urzhumtsev

**Affiliations:** a Lawrence Berkeley National Laboratory, One Cyclotron Road, BLDG 64R0121, Berkeley, CA 94720, USA; b Institute of Mathematical Problems of Biology, Russian Academy of Sciences, Pushchino 142290, Russia; c IGMBC, 1 Rue L. Fries, 67404 Illkirch and IBMC, 15 Rue R. Descartes, 67084 Strasbourg, France; d Faculty of Sciences, Nancy University, 54506 Vandoeuvre-lès-Nancy, France

**Keywords:** structure refinement, subatomic resolution, deformation density, interatomic scatterers, *PHENIX*

## Abstract

A study of the accurate electron-density distribution in molecular crystals at subatomic resolution (better than ∼1.0 Å) requires more detailed models than those based on independent spherical atoms. A tool that is conventionally used in small-molecule crystallography is the multipolar model. Even at upper resolution limits of 0.8–1.0 Å, the number of experimental data is insufficient for full multipolar model refinement. As an alternative, a simpler model composed of conventional independent spherical atoms augmented by additional scatterers to model bonding effects has been proposed. Refinement of these mixed models for several benchmark data sets gave results that were comparable in quality with the results of multipolar refinement and superior to those for conventional models. Applications to several data sets of both small molecules and macromolecules are shown. These refinements were performed using the general-purpose macromolecular refinement module *phenix.refine* of the *PHENIX* package.

## Abstract 2

Modelling deformation electron density using interatomic scatters is simpler than multipolar methods, produces comparable results at subatomic resolution and can easily be applied to macromolecules.

## 1. Introduction

The growing number of macromolecular crystals diffracting to subatomic resolution (53 models in 2003; currently 270) requires the development of appropriate methods and software to model them best. The new information obtained from such macromolecular studies has been discussed in a number of articles (see, for example, the reviews by Dauter *et al.*, 1995 ▶, 1997 ▶; Vrielink & Sampson, 2003 ▶; Petrova & Podjarny, 2004 ▶, and numerous references therein). Afonine *et al.* (2004 ▶) have shown that information about the density deformation of individual atoms can be extracted from macromolecular data at resolutions of 0.9 Å or better. As a consequence, conventional models for macromolecular structures, in which the electron density of the molecule is a simple sum of contributions from spherical atoms smeared by individual anisotropic displacements, are incomplete and provide inaccurate values for ADPs (atomic displace­ment parameters). Following previous publications, we refer to these models as IAM (independent-atom models).

Model refinement of small molecules at subatomic resolution largely uses the multipolar formalism of Hansen & Coppens (1978 ▶). For these models, the electron density is a sum of atomic contributions in which the density is no longer spherical but depends upon the chemical environment. Such a nonspherical distribution is described by a linear combination of spherical harmonics (Hansen & Coppens, 1978 ▶). Refinement of parameters of multipolar models is monitored mainly by decrease of the crystallographic *R* factor, improvement of the residual Fourier syntheses, the rigid-bond test (RBT) and other characteristics.

Lecomte and coworkers have reported a number of multipolar refinements of amino acids and nucleic acids to determine a database of multipole parameters and have described several cases of polypeptide and protein refinement using this database (for a review, see Jelsch *et al.*, 2005 ▶). Recently, the group of Coppens (Volkov *et al.*, 2007 ▶) also reported an application of the multipolar refinement to polypeptides, but using their own database of multipolar parameters.

Volkov *et al.* (2007 ▶) concluded that the applicability of multipolar models in macromolecular studies ‘is in general not warranted, unless exceptionally high-resolution data of ∼0.6 Å or better with satisfactory completeness’ are available. Also it was stated that ‘for macromolecular crystal such data are generally not available, … the number of reflections is not sufficient’. A possible solution to overcome this obstacle is a direct transfer of library parameters without their refinement as discussed by Brock *et al.* (1991 ▶), Pichon-Pesme *et al.* (1995 ▶), Jelsch *et al.* (1998 ▶), Dittrich *et al.* (2005 ▶), Volkov *et al.* (2007 ▶) and Zarychta *et al.* (2007 ▶). However, since the quality of macromolecular X-ray data is generally lower than that for small-molecule crystals, an alternative solution is to introduce a model of intermediate complexity that is more detailed than IAM but simpler than a multipolar model (Afonine *et al.*, 2004 ▶). A possible approach is to complete the IAM with spherical scatterers between the atoms (IAS, interatomic scatterers). It should be noted that the use of the IAM–IAS model is much more runtime-efficient and can be straightforwardly implemented in macromolecular crystallographic packages. Here, we use IAS instead of the previous name DBE (dummy bond electron model; Afonine *et al.*, 2004 ▶), as it better reflects the features of the model.

In this paper, we compare the results obtained with different types of electron-density models for several benchmark data sets. The implementation of IAS modelling into the general-purpose crystallographic program suite *PHENIX* (Adams *et al.*, 2002 ▶) has allowed the corresponding refinements with *phenix.refine* (Afonine *et al.*, 2005 ▶) to be performed quickly and in a fully automated fashion.

## 2. Comparative refinement at subatomic resolution

The modelling of structures at subatomic resolution with multipolar models takes into account the delocalization of electron density from atomic centres owing to the formation of interatomic bonds. The IAM–IAS model (Afonine *et al.*, 2004 ▶) instead treats this delocalized density as spherical Gaussian scatterers located at the centroid of the delocalized density and keeps the conventional spherical atoms unchanged. The multipolar model requires that existing IAM atoms be replaced, while the IAS models complete them with specifically constructed scatterers. Also, the IAM–IAS model may be gradually extended once the new features become visible. Some details of the construction and refinement of IAM–IAS models and the development of the corresponding library of parameters were originally outlined by Afonine *et al.* (2004 ▶). The current tests were aimed to demonstrate that IAM–IAS models can improve conventional IAM models by lowering the *R* and *R*_free_ factors, correcting the ADP parameters and producing clearer residual maps to the same degree as multipolar models and yet are significantly simpler to work with. In this short communication, we do not have the possibility of discussing applications other than map improvement (see, for example, Afonine *et al.*, 2002 ▶). For the same reason, the complete methodology and implementation details of IAS in *PHENIX*, including the choice of refinement targets, the role of data completeness and the efficient resolution, will be discussed separately in a full-length paper (Afonine *et al.*, in preparation).

To estimate the quality of IAM–IAS models, we built and refined such models for YGG and P2A4 (Table 1 ▶), for which a comparative refinement has been reported by Volkov *et al.* (2007 ▶). Similarly to Volkov *et al.* (2007 ▶), refinement was performed at two different resolutions. The highest available resolution (0.44 and 0.37 Å, respectively; for YGG the data completeness is below 50% at a resolution higher than 0.57 Å) was considered as ‘high resolution’, where the data-to-parameter ratio is high enough even for the use of a multipolar model, and a resolution of 0.80 Å was the ‘low resolution’ where this ratio becomes too low. In addition to the standard *R* factor and rigid-bond test (Hirshfeld, 1976 ▶), *R*_free_ (Brünger, 1992 ▶) was used as a refinement-quality indicator.

Unfortunately, the YGG and P2A4 models had been refined previously against the full set of data [in fact, the set selected with *I* > 3σ(*I*), which is not explicitly stated in Volkov *et al.*, 2007 ▶], making the conventional *R*_free_ analysis biased. Therefore, when performing the IAS refinements we only note that *R*_free_ is lower than the corresponding values for the refined IAM models.

The IAM–IAS models were generated and refined completely automatically in *PHENIX*. Table 2 ▶ shows principal refinement information. All stereochemical and ADP restraints on atomic parameters were removed for both the small molecules and macromolecules used in this study (Dauter *et al.*, 1997 ▶; Schmidt *et al.*, 2003 ▶; Petrova *et al.*, 2006 ▶). Since the starting models were previously refined to a high quality, no stereochemical distortions arising from the unrestrained refinement were observed. A decrease in the *R*_free_ shows that refinement of IAS did not overfit the experimental data and indeed improved the models. When refining at ‘low resolution’, the ADP values obtained with the IAS are smaller than those from the refinement of corresponding IAMs. Based on previous work (Afonine *et al.*, 2004 ▶; Petrova *et al.*, 2006 ▶), we believe that they are closer to the correct values of the ADPs, which will otherwise tend to increase to model the deformation density along the bonds (Coppens, 1967 ▶; Dunitz & Seiler, 1973 ▶). The rigid-bond test also confirms that the introduction of IAS improved the model. In fact, the IAS refinement with the maximum-likelihood target (Lunin *et al.*, 2002 ▶; to our knowledge never previously applied in this context) improved the models further as measured by the rigid-bond test; however, analysis of this is beyond the scope of this paper. For ‘high-resolution refinement’, mean ADP values are similar with and without IAS, as noted previously by Afonine *et al.* (2004 ▶). This indicates that the highest resolution data contain sufficient information to deconvolute the deformation-density and atomic uncertainty effects and to estimate the ADPs correctly even without IAS.

**Figure 1:**
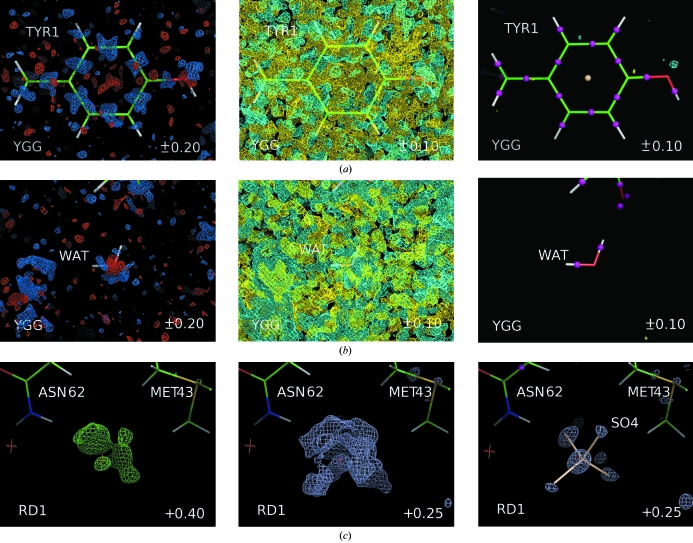
Residual Fourier maps calculated on an absolute scale. IAS are shown as small spheres in magenta (IAS with positive occupancy) and in brown (IAS with negative occupancy). (*a*, *b*) Maps at 0.43 Å resolution for YGG. Left and middle, IAM-phased maps; right, IAM–IAS-phased maps. Contour colours are +0.20 e Å^−3^ (marine), +0.10 e Å^−3^ (cyan), −0.10 e Å^−3^ (yellow) and −0.20 e Å^−3^ (red). Views are similar to those in Figs. 2 and 3 of Volkov *et al.* (2007 ▶). (*c*) Maps at 0.62 Å resolution for the antifreeze protein RD1. Left and middle, IAM-phased maps shown at cutoff levels of 0.40 e Å^−3^ (green) and 0.25 e Å^−3^ (light blue); right, IAM–IAS-phased map shown at a cutoff level of 0.25 e Å^−3^ (light blue). The sulfate ion inserted instead of the previously located water nicely fits the residual density (shown in brown).

Fig. 1 ▶ illustrates the improvement of the difference *F*_obs_ − *F*_calc_ maps, reducing the residual peaks to the same level as for multipolar models (compare with Figs. 2 and 3 in Volkov *et al.*, 2007 ▶). Overall, for the whole set of monitoring parameters the results show the comparable quality of the IAM–IAS and multipolar models despite the simplicity of the former.

Several macromolecular structures were used as another benchmark (Table 1 ▶). Previously, refinement at subatomic resolution using multipolar models has been reported for crambin (Fernandez-Serra *et al.*, 2000 ▶; 0.54 Å), trypsin (Schmidt *et al.*, 2003 ▶; 0.80 Å), phospholipase (Liu *et al.*, 2003 ▶; 0.80 Å; for resolution higher than 0.86 Å the data completeness is below 50%) and scorpion toxin (Housset *et al.*, 2000 ▶; 0.96 Å). The corresponding models were extracted from the PDB (Bernstein *et al.*, 1977 ▶; Berman *et al.*, 2000 ▶). Unfortunately, the models available in the PDB did not allow exact reproduction of the results reported, making comparative analysis of the IAS refinement impossible. In particular, this completely excluded the crambin data from our tests. To complete the picture at higher resolution, we additionally performed an IAS refinement of the antifreeze protein RD1 at 0.62 Å (Ko *et al.*, 2003 ▶). Table 2 ▶ summarizes the results of the refinement of these models. In all cases, the residual maps became much clearer. In particular, this map improvement highlighted the double conformation of the S—S bonds for the phospholipase and trypsin structures, which were otherwise hidden in the noise, and identified two ions previously interpreted as waters (Fig. 1 ▶ *c* illustrates this for RD1).

In all cases, the full round of completely automated IAS model building and IAS–IAM refinement, with no manual intervention, took from a few minutes to 1 h on a modern Linux computer. For all protein refinements, completing IAM by IAS decreases the *R* factors; the *R*_free_ factors are lower for IAS–IAM than for IAM. The RBT value systematically decreases after the introduction of IAS. We observe that the mean ADP slightly increased for the scorpion toxin data, which may indicate that the resolution (0.96 Å) approaches the limit for the use of the IAM–IAS method.

## 3. Conclusion

Currently, multipolar modelling is the most precise and powerful tool for crystallographic studies at subatomic resolution when the crystals diffract to ultrahigh resolution of about 0.6 Å or higher and the data-to-parameter ratio justifies refinement of the model parameters. At a resolution near 0.8–0.9 Å, which is more common for macromolecular crystals at sub-angstrom resolutions, multipolar modelling typically requires too many parameters to be refined. As an alternative to the multipolar method, IAM–IAS models may be used, where IAM atoms are augmented by small interatomic scatterers. This approach makes model building and refinement a very transparent and easily monitored procedure. The results of automated refinement of such models for both small molecules and macromolecules at subatomic resolution confirm the efficiency of these models both in terms of model quality and CPU resources required. The tests show that these models can be used even at ultrahigh resolution, producing results that are comparable with those obtained with multipolar models.

## Figures and Tables

**Table 1:** Data used for refinements *N*_nonH_, *N*_H_ and *N*_IAS_ give the number of non-H, H and IAS atoms in corresponding models. *d*_high_ is the highest resolution for the data set, *N*_high_ is the corresponding number of reflections. *N*_low_ is the number of reflections for the data sets truncated to lower resolution (*d*_low_ = 0.80 Å; YGG and P2A4 only).

Molecule	Space group and unit-cell parameters (Å, °)	*N*_nonH_	*N*_H_	*N*_IAS_	*d*_high_ (Å)	*N*_high_	*N*_low_	Reference
YGG	*P*2_1_2_1_2_1_, *a* = 7.98, *b* = 9.54, *c* = 18.32	22	19	39	0.43	4766	1358	Volkov *et al.* (2007 ▶)
P2A4	*P*2_1_2_1_2_1_, *a* = 10.13, *b* = 12.50, *c* = 19.50	35	36	71	0.37	21475	2513	Volkov *et al.* (2007 ▶)
Antifreeze protein (KW03)	*P*2_1_2_1_2_1_, *a* = 32.50, *b* = 39.50, *c* = 44.64	650	518	367	0.62	118501	—	Ko *et al.* (2003 ▶)
Trypsin	*P*1, *a* = 32.87, *b* = 37.02, *c* = 39.78, α = 102.89, β = 104.59, γ = 102.37	2231	1515	1362	0.80	163918	—	Schmidt *et al.* (2003 ▶)
Phospholipase	*C*2, *a* = 44.73, *b* = 59.09, *c* = 45.31, α = 90.00, β = 117.43, γ = 90.00	1324	956	679	0.80	77695	—	Liu *et al.* (2003 ▶)
Scorpion toxin	*P*2_1_2_1_2_1_, *a* = 45.90, *b* = 40.70, *c* = 30.10	647	441	335	0.96	31001	—	Housset *et al.* (2000 ▶)

**Table 2:** Comparative statistics for refinement of IAS and multipolar models M_t_ and M_r_ represent multipolar models with transferred and refined parameters (refinements ‘3’ and ‘5’ in Volkov *et al.*, 2007 ▶). 〈*B*_nonH_〉 is the mean value of the equivalent isotropic ADP calculated for non-H atoms. RBT is the rigid-bond-test value (the same as DMSDA, differences in mean-squared displacement amplitudes, in Volkov *et al.*, 2007 ▶). *R*_work_ and *R*_free_ are the standard crystallographic *R* and *R*_free_ factors between experimental *F* ^obs^ and model-based calculated structure-factor magnitudes *F* ^model^ (Afonine *et al.*, 2005 ▶) calculated as 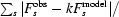 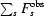.

Data set	Model	*N*_data_/*N*_par_†	*R*_work_	*R*_free_	〈*B*_nonH_〉 (Å^2^)	RBT (10^4^ Å^2^)
YGG, low resolution	IAM‡	4.9	2.16	—	—	17.76
	M_t_‡	6.2	1.22	—	—	12.85
	IAM	6.2	2.35	2.62	1.23	18.99
	IAS	4.0	1.57	2.00	1.05	12.23
YGG, high resolution	IAM‡	17.3	4.51	—	—	8.77
	M_t_‡	21.9	3.66	—	—	7.38
	M_r_‡	10.6§	3.42	—	—	6.38
	IAM	21.9	4.57	4.72	1.04	8.62
	IAS	14.2	3.75	4.06	1.07	7.68
P2A4, low resolution	IAM‡	5.5	2.98	—	—	15.64
	M_t_‡	7.1	1.84	—	—	7.09
	IAM	7.1	3.51	3.79	1.24	20.77
	IAS	4.5	2.45	3.27	1.07	16.77
P2A4, high resolution	IAM‡	46.7	3.44	—	—	3.67
	M_t_‡	61.0	2.67	—	—	2.65
	M_r_‡	43.6§	2.53	—	—	3.09
	IAM	61.1	3.72	3.63	1.14	3.66
	IAS	38.1	3.06	3.23	1.14	4.79
Antifreeze protein	IAM	18.6	12.77	15.37	7.84	208.4
	IAS	14.3	11.76	14.44	7.40	195.7
Trypsin	IAM	7.6	10.30	13.79	5.79	149.3
	IAS	5.8	9.19	13.35	5.52	126.0
Phospholipase	IAM	6.0	8.99	12.80	9.88	250.6
	IAS	4.7	8.31	12.64	9.11	213.5
Scorpion toxin	IAM	4.9	9.40	15.47	10.30	365.8
	IAS	3.9	8.78	15.23	10.42	363.1

† For multipolar refinement a number of parameters were fixed or linked by constraints. *N*_par_ is the number of parameters at each step and does not include the number of parameters refined previously. In contrast to Volkov *et al.* (2007 ▶), in the current project the ratio *N*_data_/*N*_par_ was calculated for the total number of refined parameters even when at each particular moment only a subset of them were refined; a direct comparison of this information with that reported in Volkov *et al.* (2007 ▶) is not straightforward.

‡ Refined by Volkov *et al.* (2007 ▶); corresponding numbers are cited from there.

§ An estimate obtained if the same set of parameters were used for refinement at ‘high’ resolution.

## References

[bb1] Adams, P. D., Grosse-Kunstleve, R. W., Hung, L.-W., Ioerger, T. R., McCoy, A. J., Moriarty, N. W., Read, R. J., Sacchettini, J. C., Sauter, N. K. & Terwilliger, T. C. (2002). *Acta Cryst.* D**58**, 1948–1954. 10.1107/s0907444902016657 12393927

[bb2] Afonine, P. V., Grosse-Kunstleve, R. W. & Adams, P. D. (2005). *CCP4 Newsl.* **42**, contribution 8.

[bb3] Afonine, P. V., Lunin, V. Y., Muzet, N. & Urzhumtsev, A. (2004). *Acta Cryst.* D**60**, 260–274. 10.1107/S0907444903026209 14747702

[bb4] Afonine, P. V., Pichon-Pesme, V., Muzet, N., Jelsch, C., Lecomte, C. & Urzhumtsev, A. (2002). *CCP4 Newsl.* **41**, contribution 6.

[bb5] Bernstein, F. C., Koetzle, T. F., Williams, G. J., Meyer, E. F. Jr, Brice, M. D., Rodgers, J. R., Kennard, O., Shimanouchi, T. & Tasumi, M. (1977). *J. Mol. Biol.* **112**, 535–542. 10.1016/s0022-2836(77)80200-3 875032

[bb6] Berman, H. M., Westbrook, J., Feng, Z., Gilliland, G., Bhat, T. N., Weissig, H., Shindyalov, I. N. & Bourne, P. E. (2000). *Nucleic Acids Res.* **28**, 235–242. 10.1093/nar/28.1.235 PMC102472 10592235

[bb7] Brock, C. P., Dunitz, J. D. & Hirshfeld, F. L. (1991). *Acta Cryst.* B**47**, 789–797.

[bb8] Brünger, A. (1992). *Nature (London)*, **355**, 472–475. 10.1038/355472a0 18481394

[bb9] Coppens, P. (1967). *Science*, **158**, 1577–1579. 10.1126/science.158.3808.1577 17816628

[bb10] Dauter, Z., Lamzin, V. S. & Wilson, K. S. (1995). *Curr. Opin. Struct. Biol.* **5**, 784–790. 10.1016/0959-440x(95)80011-5 8749366

[bb11] Dauter, Z., Lamzin, V. S. & Wilson, K. S. (1997). *Curr. Opin. Struct. Biol.* **7**, 681–688. 10.1016/s0959-440x(97)80078-4 9345627

[bb12] DeLano, W. L. (2002). *The PyMOL Molecular Graphics System.* DeLano Scientific, San Carlos, CA, USA. http://www.pymol.org.

[bb14] Dittrich, B., Hübschle, C. B., Messerschmidt, M., Kalinowski, R., Girnt, D. & Luger, P. (2005). *Acta Cryst.* A**61**, 314–320. 10.1107/S0108767305005039 15846034

[bb15] Dunitz, J. D. & Seiler, P. (1973). *Acta Cryst.* B**29**, 589–595.

[bb16] Fernandez-Serra, M. V., Junquera, J., Jelsch, C., Lecomte, C. & Artacho, E. (2000). *Solid State Commun.* **116**, 395–400.

[bb17] Hansen, N. K. & Coppens, P. (1978). *Acta Cryst.* A**34**, 909–921.

[bb18] Hirshfeld, F. L. (1976). *Acta Cryst.* A**32**, 239–244.

[bb19] Housset, D., Benabicha, F., Pichon-Pesme, V., Jelsch, C., Maierhofer, A., David, S., Fontecilla-Camps, J. C. & Lecomte, C. (2000). *Acta Cryst.* D**56**, 151–160. 10.1107/s0907444999014948 10666594

[bb22] Jelsch, C., Guillot, B., Lagoutte, A. & Lecomte, C. (2005). *J. Appl. Cryst.* **38**, 38–54.

[bb20] Jelsch, C., Pichon-Pesme, V., Lecomte, C. & Aubry, A. (1998). *Acta Cryst.* D**54**, 1306–1318. 10.1107/s0907444998004466 10089507

[bb21] Jelsch, C., Teeter, M. M., Lamzin, V. S., Pichon-Pesme, V., Blessing, R. H. & Lecomte, C. (2000). *Proc. Natl Acad. Sci. USA*, **97**, 31731–3176. 10.1073/pnas.97.7.3171 PMC16211 10737790

[bb23] Ko, T.-P., Robinson, H., Gao, Y.-G., Cheng, C.-H. C., DeVries, A. L. & Wang, A. H.-J. (2003). *Biophys. J.* **84**, 1228–1237. 10.1016/S0006-3495(03)74938-8 PMC1302699 12547803

[bb24] Liu, Q., Huang, Q., Teng, M., Weeks, C. M., Jelsch, C., Zhang, R. & Niu, L. (2003). *J. Biol. Chem.* **278**, 41400–41408. 10.1074/jbc.M305210200 12871974

[bb25] Lunin, V. Y., Afonine, P. V. & Urzhumtsev, A. G. (2002). *Acta Cryst.* A**58**, 270–­282. 10.1107/s0108767302001046 11961289

[bb26] Petrova, T., Ginell, S., Mitschler, A., Hazemann, I., Schneider, T., Cousido, A., Lunin, V. Y., Joachimiak, A. & Podjarny, A. (2006). *Acta Cryst.* D**62**, 1535–1544. 10.1107/S0907444906041035 17139089

[bb27] Petrova, T. E. & Podjarny, A. D. (2004). *Rep. Prog. Phys.* **67**, 1565–1605.

[bb28] Pichon-Pesme, V., Lecomte, C. & Lachekar, H. (1995). *J. Phys. Chem.* **99**, 6242–6250.

[bb29] Schmidt, A., Jelsch, C., Østergaard, P., Rypniewski, W. & Lamzin, V. S. (2003). *J. Biol. Chem.* **278**, 43357–43362. 10.1074/jbc.M306944200 12937176

[bb30] Volkov, A., Messerschmidt, M. & Coppens, P. (2007). *Acta Cryst.* D**63**, 160–­170. 10.1107/S0907444906044453 17242509

[bb31] Vrielink, A. & Sampson, N. (2003). *Curr. Opin. Struct. Biol.* **13**, 709–715. 10.1016/j.sbi.2003.10.012 14675549

[bb32] Zarychta, B., Pichon-Pesme, V., Guillot, B., Lecomte, C. & Jelsch, C. (2007). *Acta Cryst.* A**63**, 108–125. 10.1107/S0108767306053748 17301471

